# Nature’s endless wonder: unexpected motherhood after pediatric allogeneic stem cell transplantation and severe late effects

**DOI:** 10.1007/s00508-020-01642-9

**Published:** 2020-04-07

**Authors:** Dorothea Bauer, Raffaela Tüchler, Daniela Dörfler, Anita Lawitschka

**Affiliations:** 1 St. Anna Children’s Hospital, SCT-Outpatient&Aftercare Clinic, Medical University Vienna, Kinderspitalgasse 15, 1090 Vienna, Austria; 2 Medical University Vienna, Vienna, Austria; 3 Department of Gynecology and Obstetrics, Medical University of Vienna, Vienna, Austria

**Keywords:** Fertility, Pregnancy, Late effects, Hypogonadism, Chronic graft versus host disease

## Abstract

Infertility and endocrine late effects (LE) are common sequelae after pediatric allogeneic hematopoietic stem cell transplantation (HSCT) after myeloablative conditioning. Nevertheless, the individual risk for these LE is not always easy to predict and therefore these issues are of ongoing interest to the clinical research community dealing with HSCT aftercare. This article describes the case of a young woman who received polychemotherapy and total body irradiation (TBI) containing conditioning for HSCT for a relapsed anaplastic large cell lymphoma (ALCL). She developed severe sclerotic chronic graft-versus-host disease (GVHD) with irreversible joint contractures and multiorgan involvement, requiring long-term multimodal immunosuppressive treatment. Subsequently showing a considerable number of LE including hypergonadotropic hypogonadism, she accepted that infertility would be quite likely. Her courageous personal life planning included part-time working and a partnership but not motherhood. This article reports the unexpected and spontaneous pregnancy and the extreme preterm birth of a surprisingly adequately developing child.

## Introduction

Gonadal dysfunction and infertility are common late effects (LE) after pediatric allogenic hematopoietic stem cell transplantation (HSCT) in females [1]. The incidence of endocrine LE is reported to be higher in chronic graft-versus-host disease (cGVHD) patients, mainly due to long-term immunosuppressive therapy [2]. Since the production of germ cells is completed in the postnatal ovary, follicular depletion caused by adverse effects of cancer treatment or conditioning therapy may be definite [3]. This can result in ovarian failure or in early onset of menopause [4].

The risk of developing gonadal failure is decisively influenced by the pubertal stage at HSCT (before or after onset of puberty) and by the conditioning regimen [5]. Although the ovaries of prepubertal children and adolescents are relatively resistant to chemotherapy-induced damage compared to the ones of adults, alkylating agents such as cyclophosphamide (CY) or high-dose busulfan (BU) are considered to impair gonadal function even in very young patients [1, 6]. Total body irradiation (TBI) as conditioning for HSCT shows the greatest risk of evoking acute ovarian failure. Among girls being treated with fractionated TBI, prepubertal girls are more likely to maintain ovarian function, [7] but still 50% of girls being aged under 10 years at the time of transplantation will suffer acute loss of ovarian function [8]. Ovarian dysfunction, especially when observed early after HSCT, may be transient [9]. Persistent hypergonadotropic hypogonadism during long-term follow-up, however, is often an indication of permanent ovarian failure. Nonetheless, rare cases of spontaneous recovery of ovarian function many years after onset have been reported [10].

If pregnancies occur in patients after HSCT, they are likely to have a successful outcome; however, they carry higher risks for spontaneous abortions and miscarriages, preterm delivery and low birth weight. Thus, these pregnancies should be treated as high risk for maternal and fetal complications [11].

## Case presentation

**Fig. 1:**
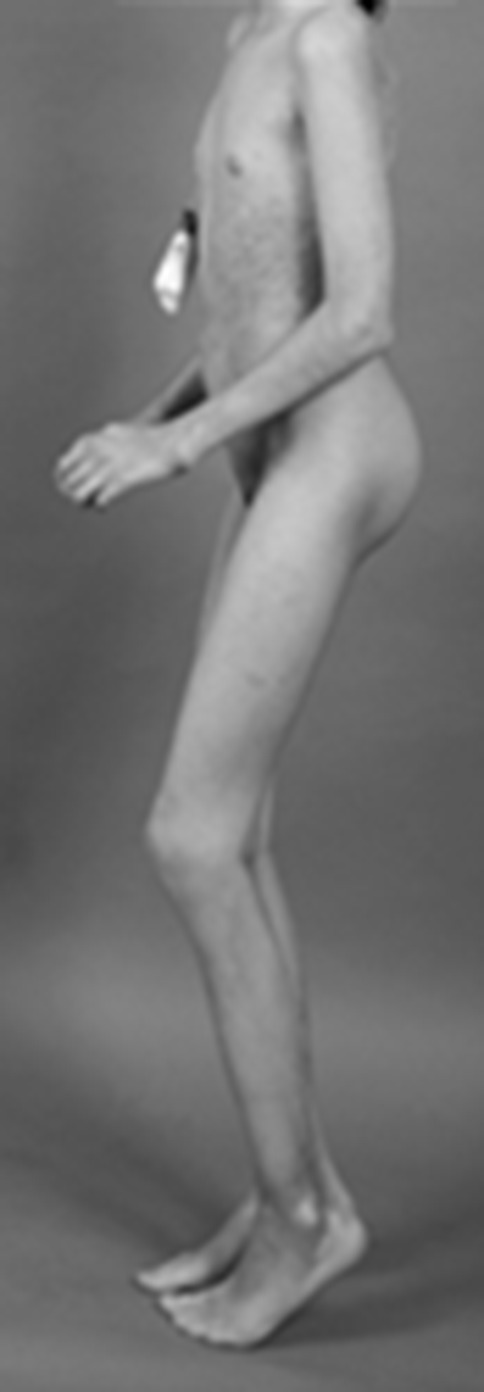
The patient in January 2007 (14 years old) showing severe cachexia, scleroderma and joint contractures

The patient, born in March 1993, was diagnosed with an anaplastic large cell lymphoma (ALCL) in October 2002 and received polychemotherapy according to the international ALCL 99 protocol: standard risk arm (cytarabine, ifosfamide, methotrexate, etoposide, doxorubicin) [12], resulting in complete remission. When a relapse occurred in January 2003 polychemotherapy according to NHL-BFM 95 was initiated [13]. After three cycles of chemotherapy (including vindesine, cytarabine, etoposide, methotrexate and ifosfamide) tumor progression was diagnosed in March 2003 but luckily after treatment intensification with vincristine, cyclophosphamide, methotrexate, doxorubicin, vindesine, cytarabine and etoposide tumor regression could be achieved. The indications for an allogenic HSCT to ensure long-term cure were confirmed. The myeloablative conditioning consisted of fractionated TBI (12 Gy), etoposide and cyclophosphamide. As GVHD prophylaxis methotrexate (MTX) and cyclosporin A (CSA) were administered. The HSCT with bone marrow of a matched family donor was performed in May 2003 and the patient has been in remission since; however, she developed extensive multisystemic sclerotic cGVHD including scleroderma, joint contractures, keratoconjunctivitis sicca, xerostomia, vulvovaginitis, chest wall sclerosis with a combined ventilation disorder, which made a multimodal immunosuppressive treatment in several treatment lines over 6 years necessary. Subsequently, she developed malabsorption and cachexia, a reactive depressive disorder and osteoporosis, which complicated her long-lasting time of rehabilitation and psychosocial reintegration. Finally, immunosuppressive treatment could be tapered and stopped in 2009, leaving partial scleroderma and joint contractures with reduced range of motion as irreversible but inactive GVHD sequelae. Movement in all joints was reduced, especially in both wrist joints, where no flexion, hardly any extension and no rotation were possible. Most movements in the fingers were limited. Movement in the hips (abduction/adduction right: 30°-0°-40°, left: 30°-0°-20°) and knees (extension/flexion left and right: 10°-0°-135°) were partially reduced (Fig. 1).

In 2007 she was diagnosed with delayed puberty connected with primary amenorrhea and hypergonadotropic hypogonadism. Hormonal replacement therapy (HRT) with estrogen for puberty induction was slowly initiated and increased to adult dosing over 2 years. When breakthrough bleeding occurred, progesterone was added based on the clinical practice guidelines for the care of girls and women with Turner syndrome [14]. Hormone levels were frequently checked with no substantial change for 7 years. In 2010 HRT withdrawal was tried for 3 months, still showing reduced gonadal function. After successful pubertal development, HRT was switched to a combination of progestin and estradiol in 2013. In 2015 HRT was discontinued at low follicle stimulating hormone (FSH) and luteotropic hormone (LH) levels and 6 months later (beginning of year 2016, at the age of 22 years) the patient reported a normal menstrual cycle for the first time in her life. She reported regular bleeding until December 2016. Antimüllerian hormone (AMH) was not detectable (<0.08 ng/ml, Table 1).

**Table 1:** Hormone levels in the patient before and after pregnancy

Hormone	Before pregnancy^a^	After pregnancy^b^	Reference range	Unit
Luteotropic hormone	2.6	4.7	2.4–95.6	mlU/mL
Follicle stimulating hormone	4.5	5.6	1.7–21.5	mlU/mL
17-beta estradiol	<5	89	26.7–314	pg/mL
Parathyroid hormone	24.1	25.5	15–65	pg/mL
25-hydroxy-vitamin D	109.0	44.9	75–250	nmol/L
Osteocalcin	21.0	21.4	11–36	ng/mL
Antimüllerian hormone	<0.08	0.10	0.89–9.85	ng/mL

^a^ 2015/2016

^b^ 2018

According to international guidelines, the patient was cared for in the St. Anna HSCT outpatient and LE clinic by an interdisciplinary team including rehabilitation stays, regular psychological and physiotherapeutic care, professional and nutritional counselling, social work and occupational therapy [2, 15]. She was able to complete an apprenticeship as an office administrator and then worked part-time.

On January 2017, at the age of 23 years, the unexpected pregnancy at an estimated 19 + 4 weeks gestational age and cervical incompetence was diagnosed during a routine control at the Department of Gynecology of Vienna General Hospital. The patient was hospitalized for 2 days and daily treatment with 200 mg progesterone vaginally was started. The patient was rehospitalized at 21 + 0 weeks gestational age because of progressive cervical incompetence. She received a tocolytic therapy (hexoprenaline) and an antibiotic prophylaxis. At 23 + 4 gestational age fetal lung maturity was induced as premature rupture of membranes occurred and the child was delivered spontaneously 2 days later at 23 + 6 weeks gestational age. Despite hip abduction being reduced due to joint contractures, spontaneous birth was possible. The boy’s weight was 620 g, with a body length of 31.5 cm, 22.5 cm circumference of the head, APGAR score 8/8/9, Natrium-pH (NA-pH) 7.27 and Base-excess (BE) −1 mmol/l. He was breathing spontaneously but due to respiratory distress syndrome and extreme immaturity he had to be immediately transferred to the neonatological intensive care unit. A string of diagnoses followed, the most severe being the extremely low birthweight, apnea and bradycardia, patent ductus arteriosus, intraventricular hemorrhage (IVH) grade 1, anemia, hypertension and retinopathia praematorium (ROP) grade 2. On day 8 the infant suffered a respiratory failure and had to be intubated for 12 days. Over the following 4 months of hospitalization most of the initial diagnoses significantly improved. After 6 weeks the cranial ultrasound showed that the IVH grade 1 was reabsorbed, and the boy could get off respiratory support after 3 months, being discharged with medication for high blood pressure, a home monitor, weighing 3844 g. The following check-ups showed further improvement of the ROP and the neurological development was appropriate for the medical history.

Maternal complications included a grade 3 perineal tear and an episiotomy (which were treated surgically) and placental retention, which required manual removal of the placenta. The grade 3 perineal tear and the necessity of an episiotomy in the birth of a child weighing only 620 g were most likely due to the mother’s scleroderma, which leaves the skin extremely rigid. The patient could be dismissed from hospital 11 days after giving birth.

The latest hormone status in May 2018 is summarized in Table 1. Postpartum, the patient returned to a regular menstrual cycle and does not need any HRT. She reports a satisfactory sexual life and no problems concerning continence. Particularly striking is the AMH level of 0.1 ng/ml which has risen compared to the last value before the pregnancy.

## Discussion

The present case history is sensational in several aspects: firstly, recovery of ovarian function and thus spontaneous conception is extremely rare in female childhood cancer survivors having been diagnosed with hypogonadism for several years [16, 17].

Rare cases of unexpected recovery of ovarian function many years after HSCT (and in some cases after TBI-based conditioning regimens) have been published, however, none of them reporting pregnancy [10, 18].

Borgmann-Staudt et al. investigated the long-term fertility outcome of 344 patients after pediatric HSCT. Successful pregnancies were reported in three females, none of them having received TBI [19]. Details on the hormone status of these patients after HSCT were not given in this study; however, the median follow-up was 6 years, indicating shorter intervals of hypogonadism as in the present case.

It is not only surprising that the patient became pregnant after having been diagnosed with hypogonadism for 7 years, but also that she showed non-detectable AMH levels (<0.08 ng/ml) at the last check-up before pregnancy. In the general population, this would not only be considered to be too low to conceive spontaneously, but is also far below the established cut-off value of 1.4 ng/ml to predict an ongoing pregnancy after assisted reproduction [20]. Several studies showed decreased AMH levels among female survivors of childhood cancer. Especially in patients reporting normal menses, AMH measurement may be a useful tool to search for subfertility [21]. Among those having received three or more cycles of polychemotherapy or TBI, AMH levels are reported to be the lowest which both polychemotherapy and TBI applies to our patient; however, for female patients with a history of pediatric HSCT and pathologic AMH values, the correlation between especially low values and fewer pregnancy rates has not been described so far.

Of further research interest would be the long-term evaluation of AMH serum levels after pediatric HSCT. In the present case, AMH levels increased 15 years after HSCT at the age of 25 years from undetectable values to 0.1 ng/ml. Miyoshi et al. published 2016 observations on AMH levels in three girls after cancer treatment: two of them received HSCT; in one patient AMH levels became undetectable during follow-up and in the other AMH levels recovered only transiently; however, follow-up was only 36 months in this study [22].

A prospective pilot study was recently conducted at the SCT aftercare clinic of the St. Anna Children’s Hospital in cooperation with the Outpatient Clinic for children and adolescent gynecology of the University Clinic for Gynecology Vienna on gynecologic aftercare and the human papilloma virus status of females aged 12–22 years (submitted). Hypogonadism was observed in 67% (12/18) of the patients and pathologic AMH levels in 94% (17/18).

Secondly, female survivors of childhood cancer are less likely to have a live birth than healthy siblings [23].

Belashov et al. published in 2011 a case report about a pregnancy in a patient after multiple courses of high-dose chemotherapy and BU-based myeloablative conditioning for HSCT. Ovarian recovery occurred already 1 year after HSCT and the pregnancy ended in an abortion at 11 weeks gestational age.

In the present case, risk factors for the progress of the pregnancy included not only the history of multiple courses of high-dose polychemotherapy, TBI and HSCT, but also the development of an extensive multisystemic sclerotic cGVHD including chest and abdominal wall sclerosis requiring long-term multimodal immunosuppressive treatment. For patients with scleroderma, higher risks of pre-eclampsia, premature delivery and small full-term infants are reported [24]. In the placentas, severe fibrosis and abnormal vascular remodeling were detected. This may result in reduced blood flow leading to damages of the maternal placenta and possible premature delivery [25]. There are no published data on pregnancy outcomes in patients with severe multisystem sclerotic cGVHD.

Thirdly, nearly 40% of infants born at 24 weeks gestational age do not survive and another 40% show severe neurodevelopmental impairment [26]. Thus, also the favorable outcome of the child is considered to be extraordinary. Due to the late diagnosis of pregnancy it appears that the child was small for gestational age and older than expected. This would also explain the favorable outcome.

## Conclusion

To our knowledge this is the first published case of ovarian function restoration, spontaneous pregnancy and birth of a ultimately adequately developed child in a female patient having been diagnosed with hypogonadism for 7 years after multiple courses of high-dose poly-chemotherapy, TBI-based myeloablative conditioning for pediatric HSCT followed by severe sclerotic cGVHD and multiple LE.

This case proves that recovery of ovarian function is possible even many years after the onset of primary ovarian failure and it illustrates the complexity of diagnosis and outcome prediction of ovarian malfunction and infertility after pediatric HSCT.
